# Supplementary material for: Defects and nanostrain gradients control phase transition mechanisms in single crystal high-voltage lithium spinel
Source: Nat Commun. 2023 Nov 1;14:6975. doi: 10.1038/s41467-023-42285-4 (PMC10620135; doi:10.1038/s41467-023-42285-4)
Supplement: Supplementary file 1 — Supplementary information [file 41467_2023_42285_MOESM1_ESM.pdf]

Supplementary Information: Defects and nanostrain gradients control phase transition mechanisms in single crystal lithium spinel

Isaac Martens,† Nikita Vostrov,† Marta Mirolo,† Steven Leake,† Edoardo Zatterin,† Xiaobo Zhu,‡ Lianzhou Wang,¶ Jakub Drnec,† Marie-Ingrid Richard,*,†,§ and Tobias U. Schulli*,†

†*European Synchrotron Radiation Facility, 71 Avenue des Martyrs, Grenoble 38000, France*

‡*College of Materials Science and Engineering, Changsha University of Science and Technology, Changsha 410114, China*

¶*Nanomaterials Centre, School of Chemical Engineering, and Australian Institute of Bioengineering and Nanotechnology, University of Queensland, QLD 4072 Australia*

§*Univ. Grenoble Alpes, CEA Grenoble, IRIG, MEM, NRX, 17 rue des Martyrs 38000 Grenoble, France*

E-mail: mrichard@esrf.fr; schulli@esrf.fr

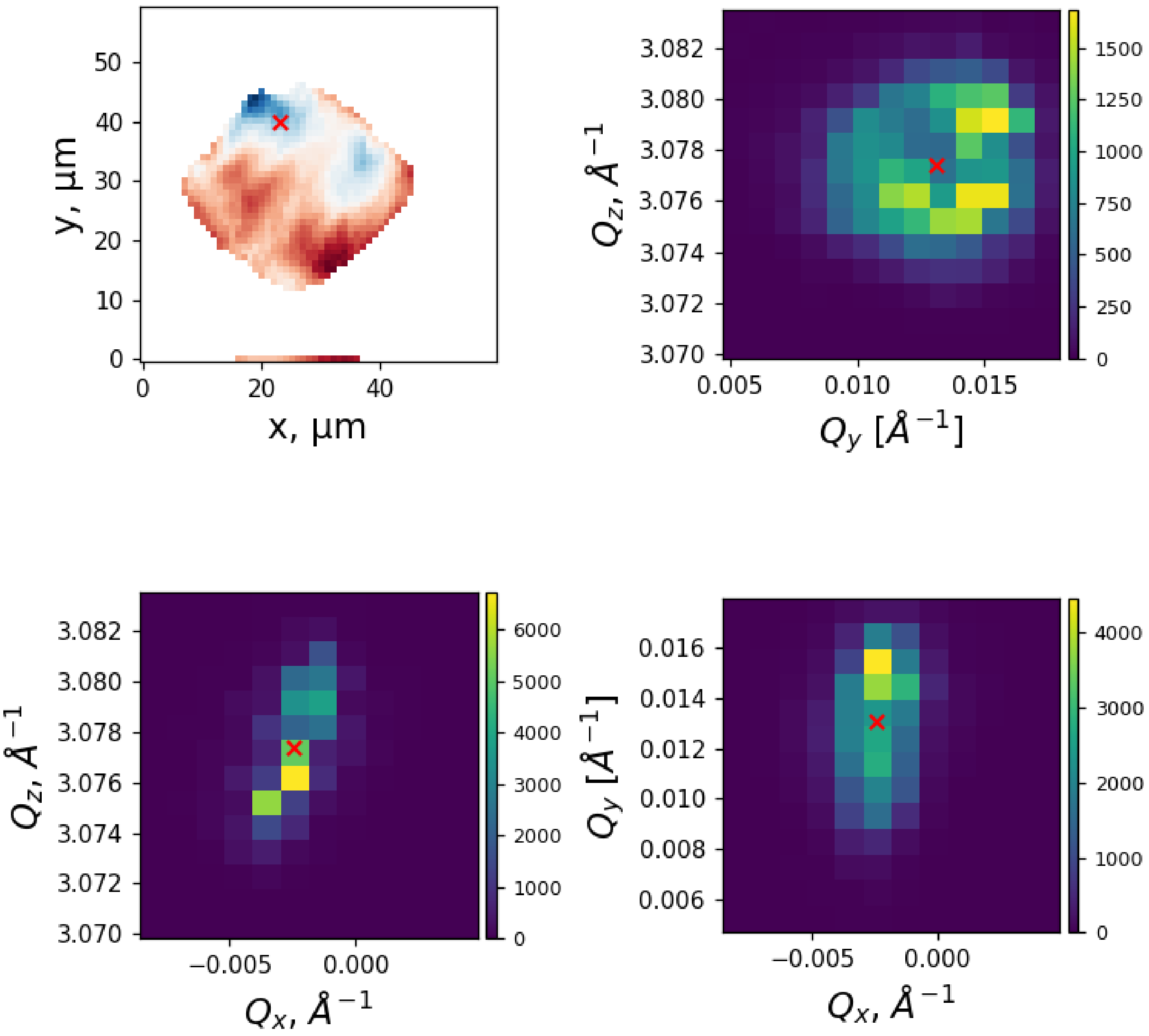

Supplementary Figure 1: (a) Integrated intensity of the measured particle. (b-d) Slices through the reciprocal space. The red cross overlaid on the integrated intensity (a) indicates the position where the measured slices were collected. Similarly, the red crosses on the slices (b-d) in reciprocal space represent the position of the scattering vector, $\mathbf{Q}_{400}$.

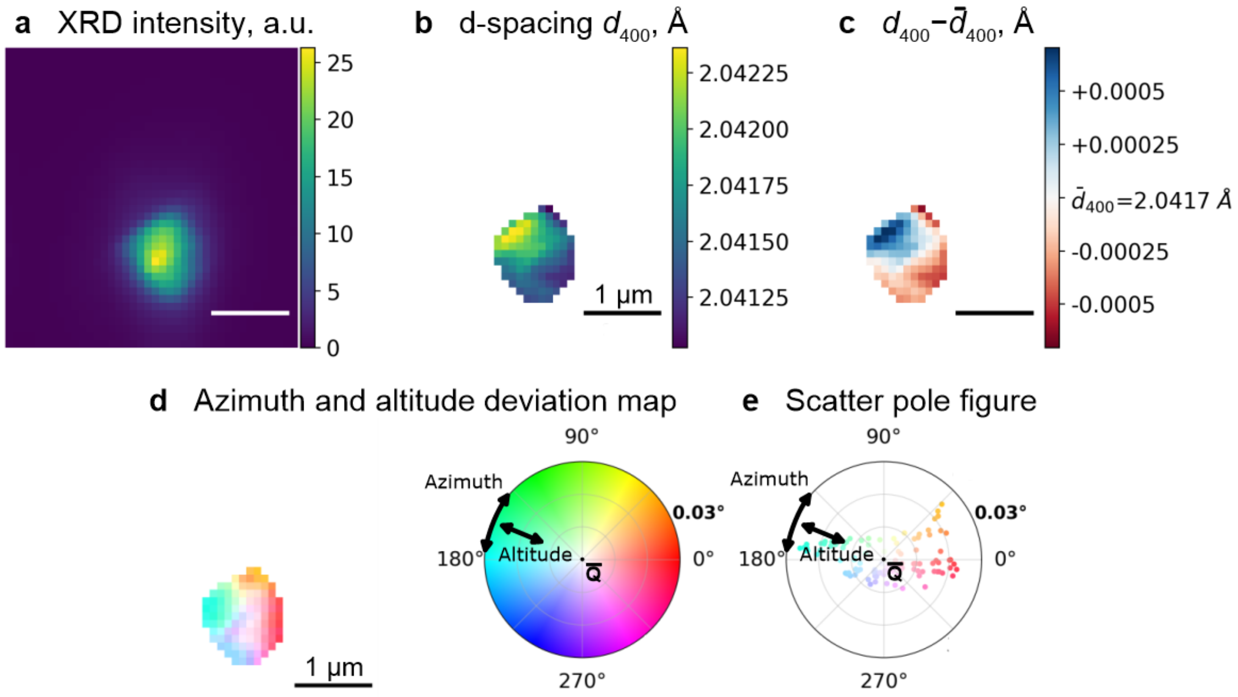

Supplementary Figure 2: *Ex situ* nanodiffraction imaging on a LMNO single crystal. Various parameters were calculated for each pixel in the map, including the scattering intensity (a), the d-spacing between the (400) planes (b), the variation of the d-spacing from its average value across the whole particle (c), the azimuth and altitude of the tilt vector relative to the average orientation of the crystal (d). The angular tilts for each pixel can be visualized as a pole figure (e).

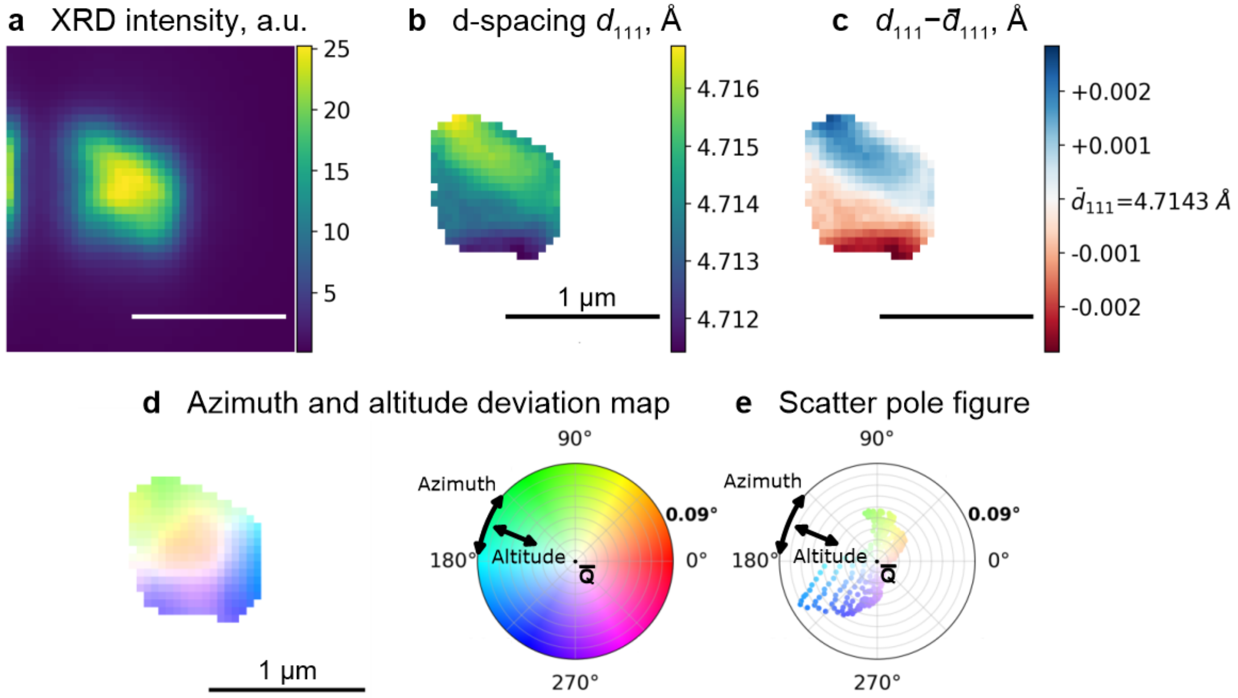

Supplementary Figure 3: *Ex situ* nanodiffraction imaging on a LMNO single crystal. Various parameters were calculated for each pixel of the map, including the scattering intensity (a), the d-spacing between the (111) planes (b), the variation of the d-spacing from its average value across the whole particle (c), azimuth and altitude of the tilt vector relative to the average orientation of the crystal (d). The angular tilts for each pixel can also be visualized as a pole figure (e).

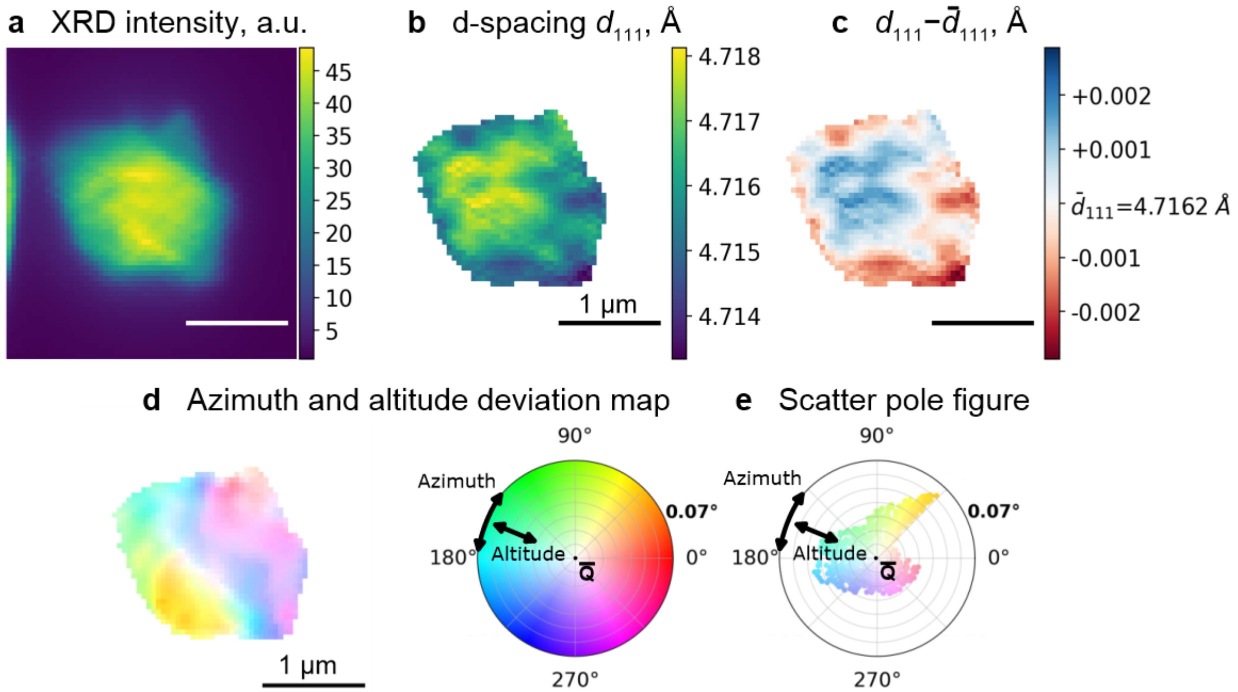

Supplementary Figure 4: *Ex situ* nanodiffraction imaging on a LMNO single crystal. Various parameters were calculated for each pixel of the map, including the scattering intensity (a), the d-spacing between the (111) planes (b), the variation of the d-spacing from its average value across the whole particle (c), azimuth and altitude of the tilt vector relative to the average orientation of the crystal (d). The angular tilts for each pixel can also be visualized as a pole figure (e).

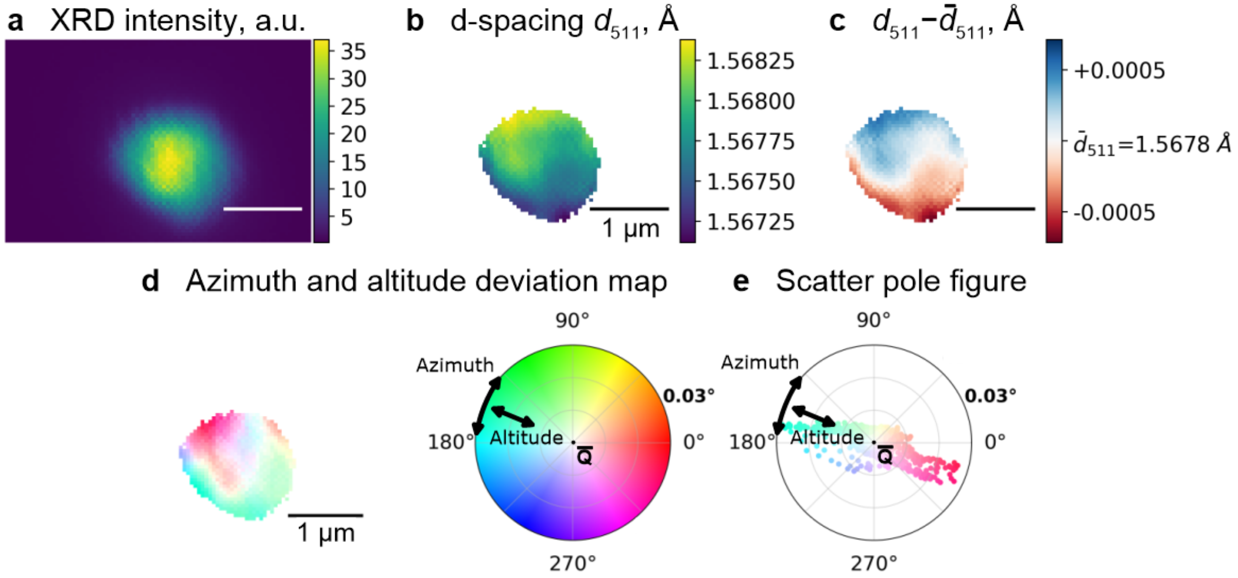

Supplementary Figure 5: *Ex situ* nanodiffraction imaging on a LMNO single crystal. Various parameters were calculated for each pixel of the map, including the scattering intensity (a), the d-spacing between the (111) planes (b), the variation of the d-spacing from its average value across the whole particle (c), azimuth and altitude of the tilt vector relative to the average orientation of the crystal (d). The angular tilts for each pixel can also be visualized as a pole figure (e).

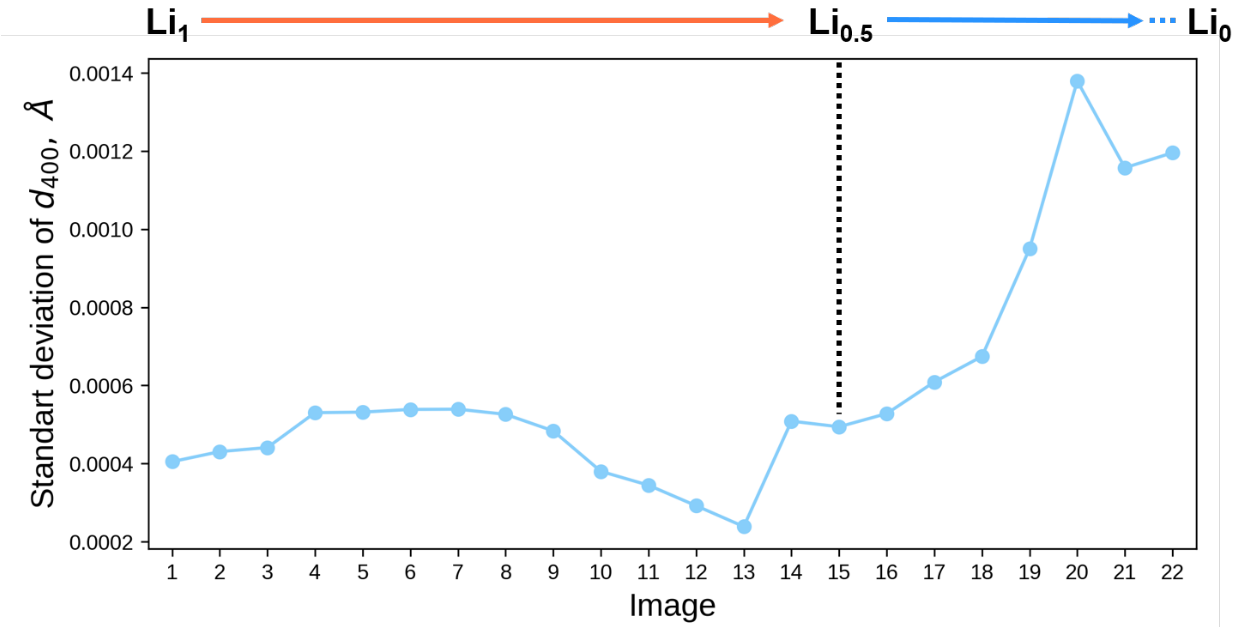

Supplementary Figure 6: Evolution of the standard deviation of the d_{400}-spacing during charging.

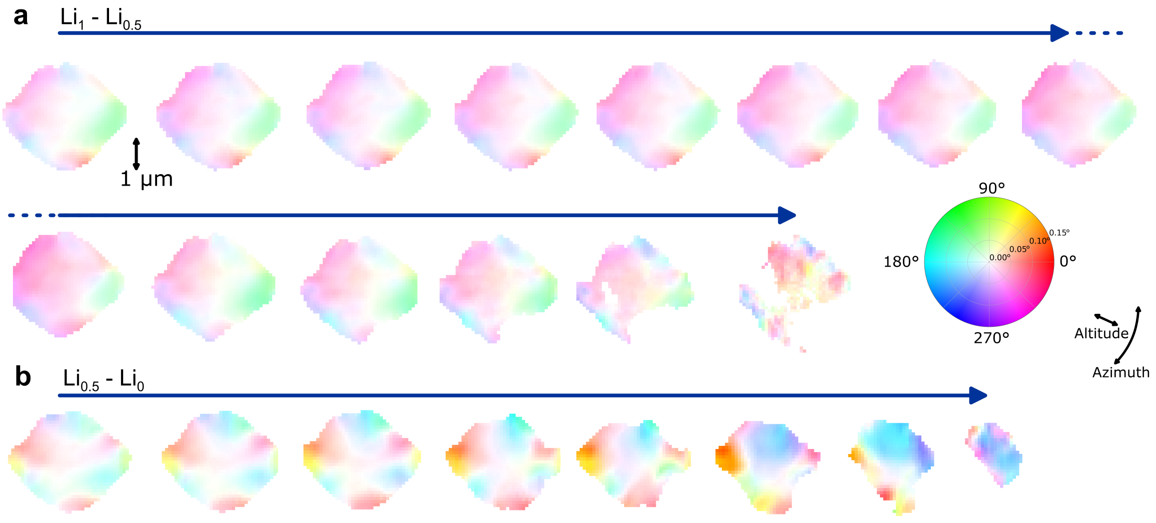

Supplementary Figure 7: Misorientation maps for all SXDM images, displayed on a unified color scale.

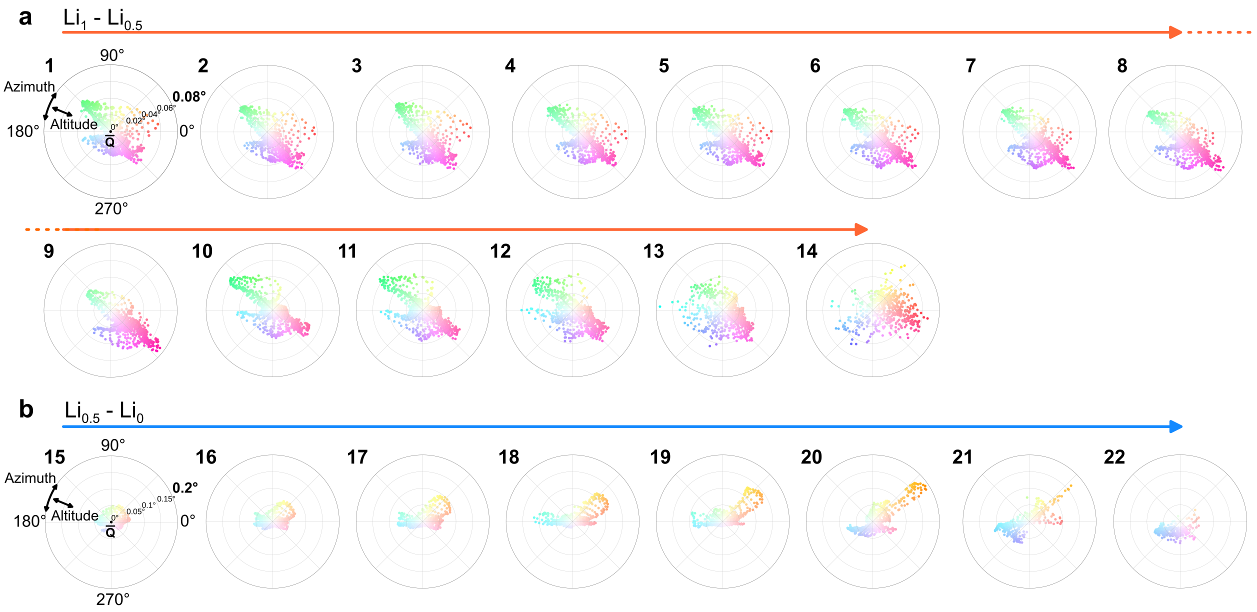

Supplementary Figure 8: Pole figure scatter plots for all SXDM images, where each point represents the azimuth and altitude deviation of the local $\mathbf{Q}_{004}$ vector at each pixel of the map.

9 16 20 with std devs.png

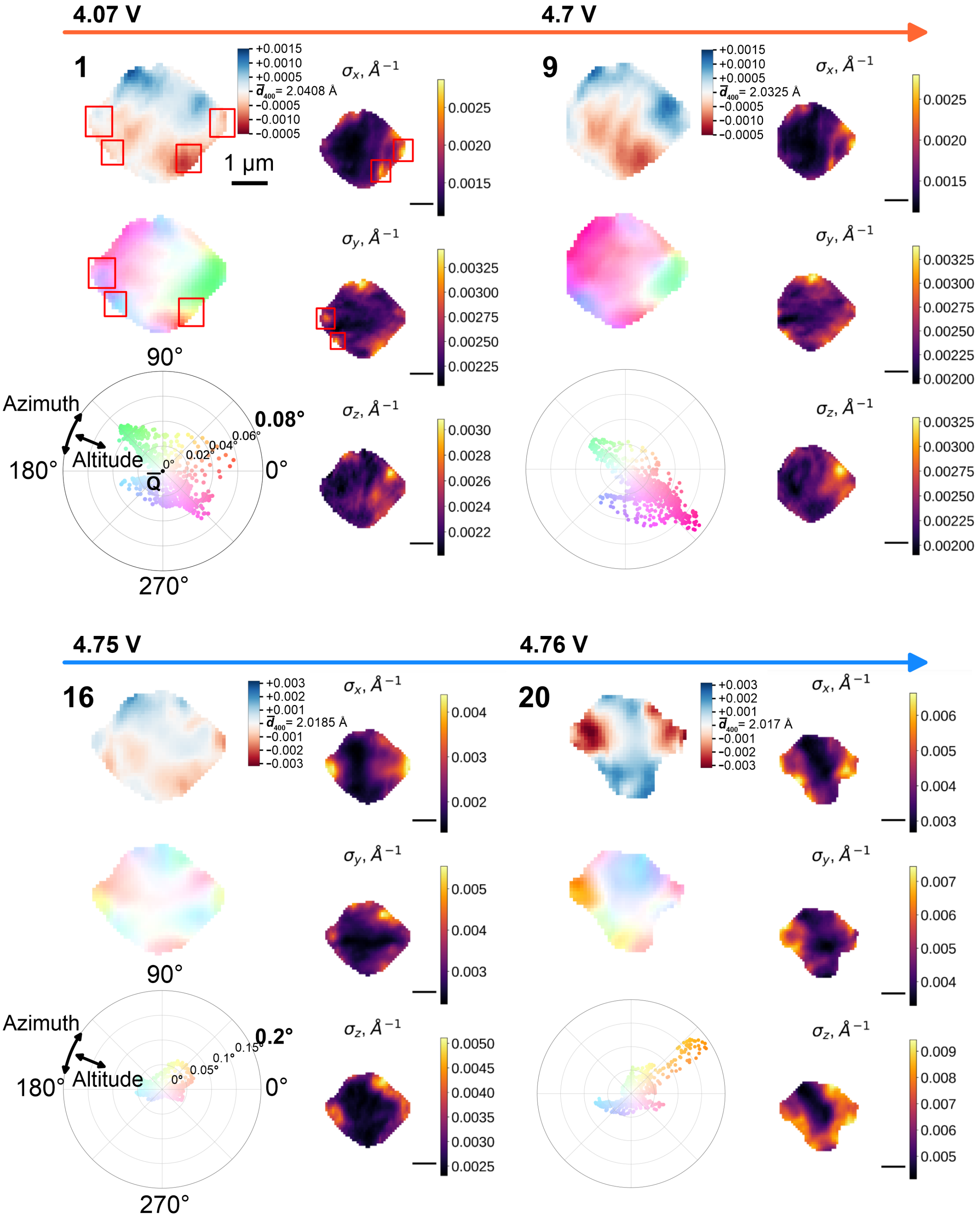

Supplementary Figure 9: Full set of maps obtained during a series of SXDM scans (including d-spacing map, misorientation map, pole figure and maps of the standard deviation for x, y and z axes of the reciprocal space) for both beginning and end of the $Li_1 \longrightarrow Li_{0.5}$ transition (scan number 1 and 9) and beginning and end of the $Li_{0.5} \longrightarrow Li_0$ transition (scan number 16 and 20). The limits of the colorbars are set to the maximum and minimum values observed during each of the phase transitions.

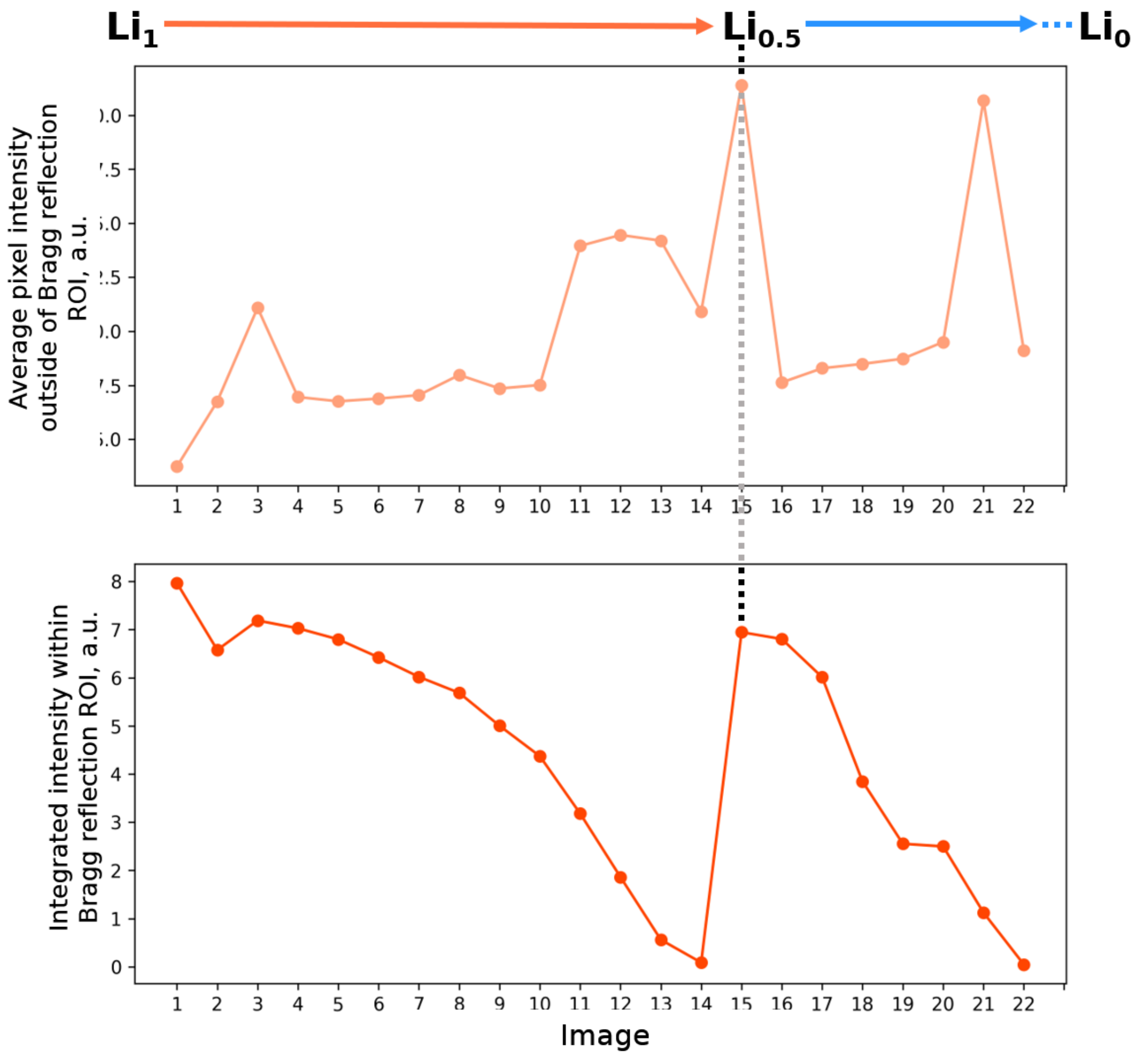

Supplementary Figure 10: Evolution of the average intensity inside the voxels of the reciprocal space volume that are located outside of the region of interest (ROI) containing the Bragg reflection (top) and integrated intensity within the aforementioned ROI (bottom).

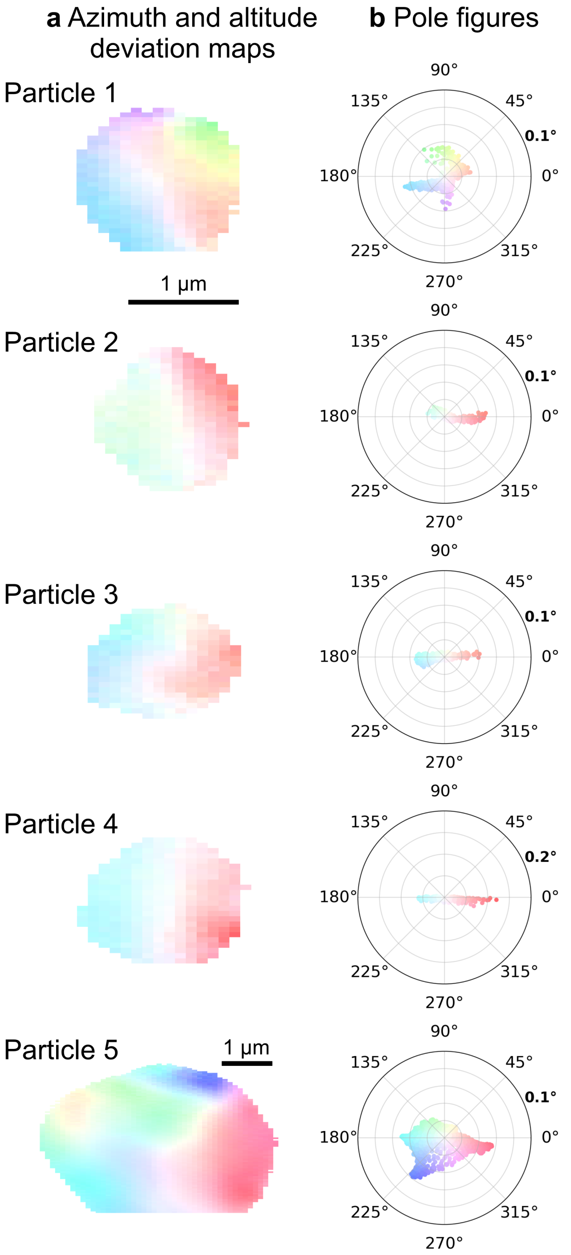

Supplementary Figure 11: Misorientation maps of pristine LMNO samples.

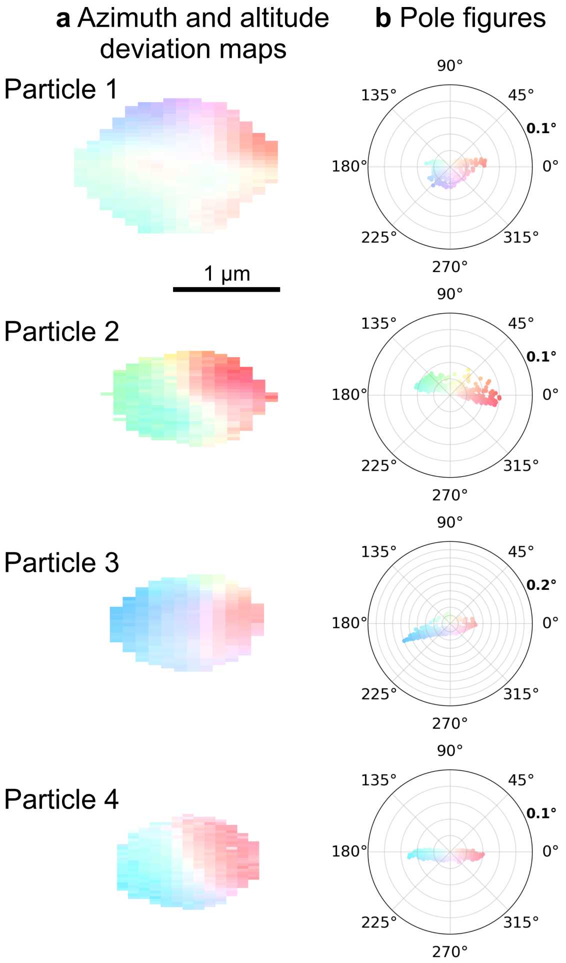

Supplementary Figure 12: Misorientation maps of cycled LMNO samples (60 cycles).

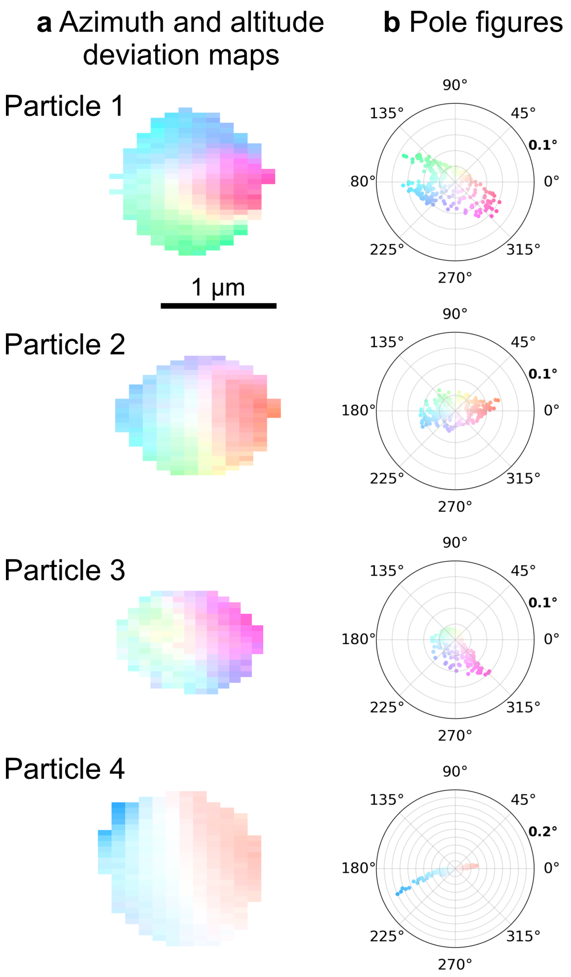

Supplementary Figure 13: Misorientation maps of cycled LMNO samples (90 cycles).

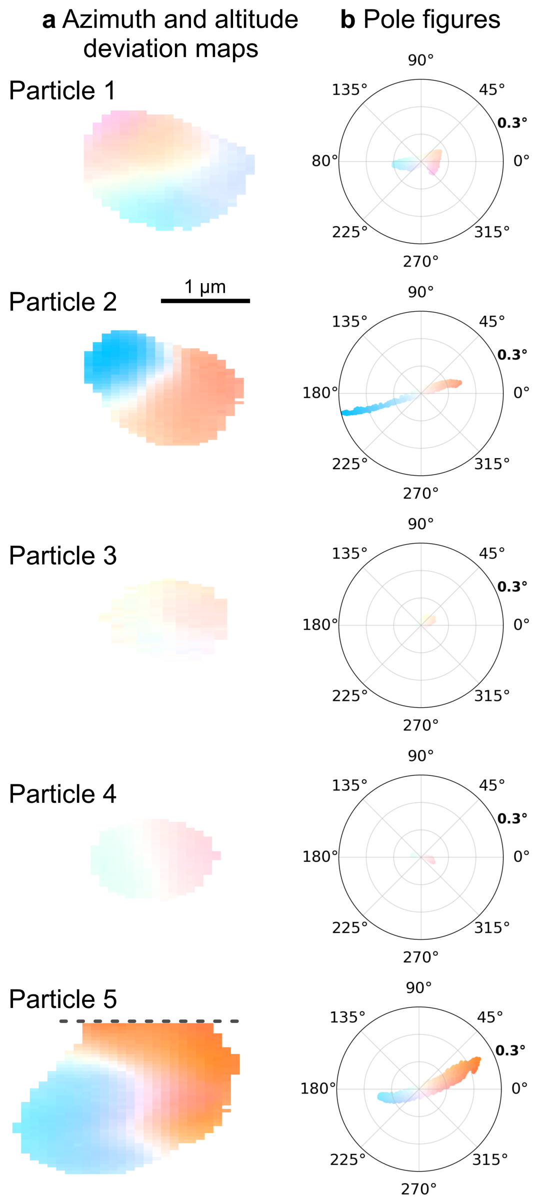

Supplementary Figure 14: Misorientation maps of cycled LMNO samples (275 cycles).

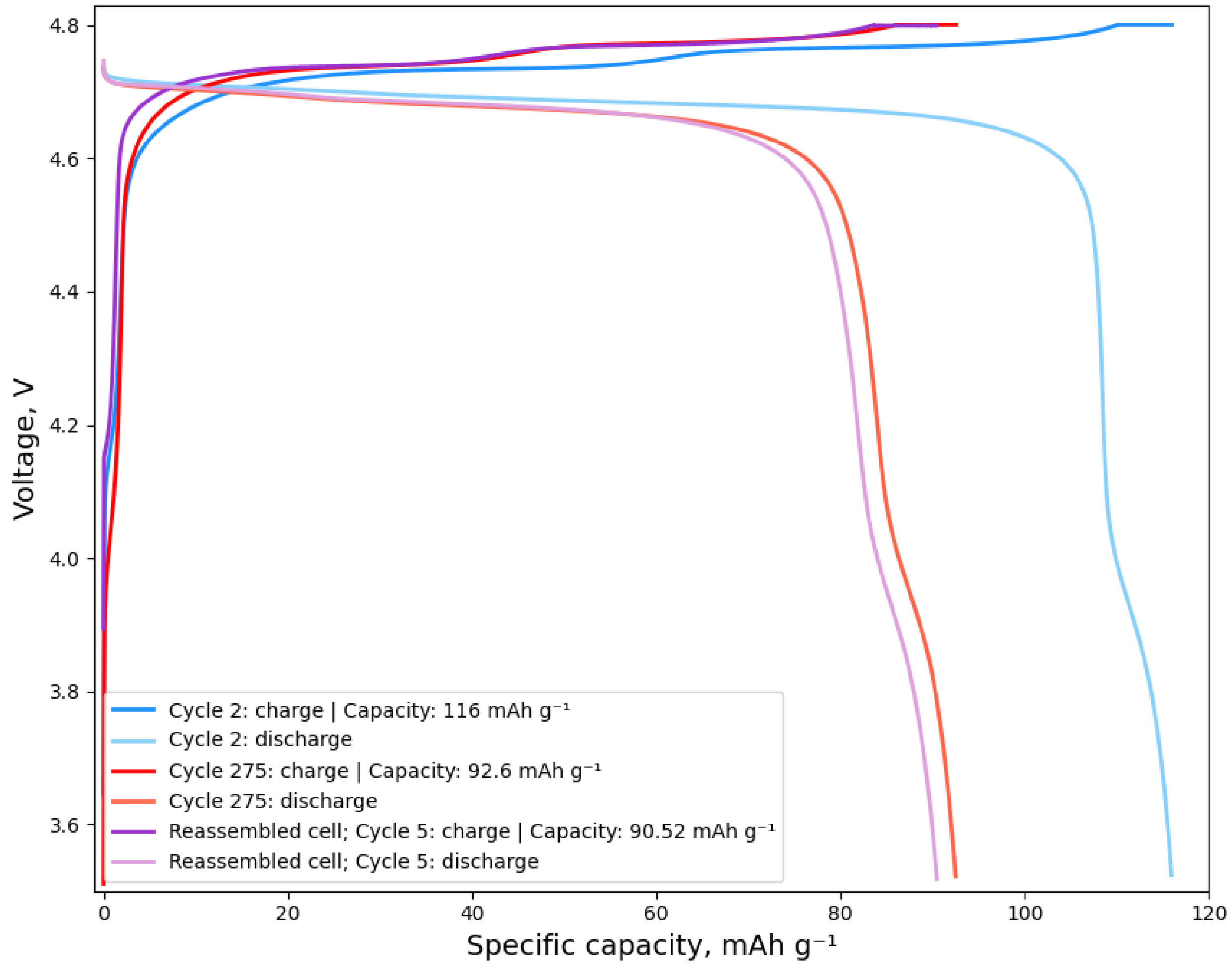

Supplementary Figure 15: Electrochemical charge and discharge curves for the cycles 2 (red) and 275 (blue) for one on the long-term cycling coin-cells. The purle curves correspond to the coin-cell reassembled with new electrolyte and Li anode but using the same LMNO cathode as the previous cell.

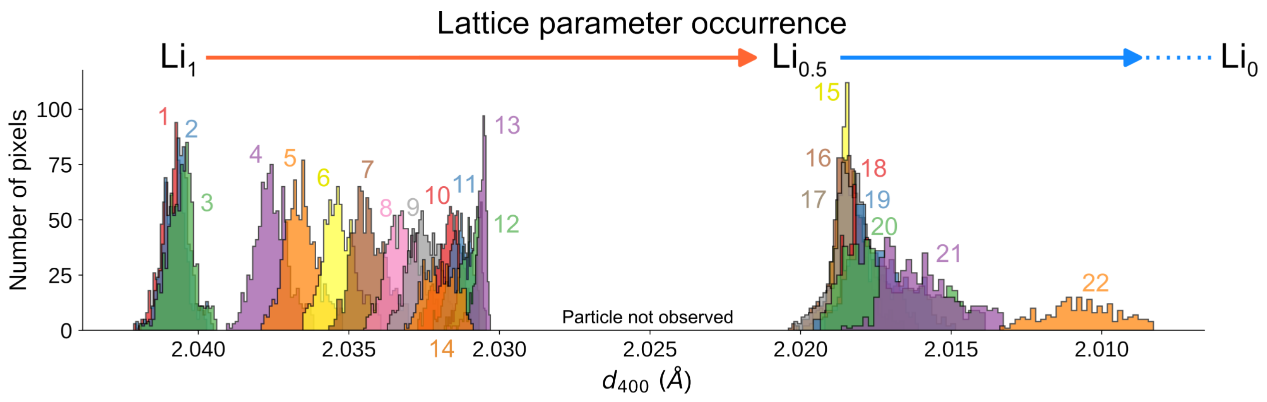

Supplementary Figure 16: Evolution of the d_{400} histogram of the particle during charging.
